# Promising Antifungal Targets Against *Candida albicans* Based on Ion Homeostasis

**DOI:** 10.3389/fcimb.2018.00286

**Published:** 2018-09-04

**Authors:** Yiman Li, Licui Sun, Chunyan Lu, Ying Gong, Min Li, Shujuan Sun

**Affiliations:** ^1^School of Pharmaceutical Sciences, Shandong University, Jinan, China; ^2^Department of Pharmacy, Feicheng Mining Central Hospital, Feicheng, China; ^3^Department of Pharmacy, Qianfoshan Hospital Affiliated to Shandong University, Jinan, China

**Keywords:** *Candida albicans*, antifungal targets, ion homeostasis, virulence, ion signaling pathways

## Abstract

In recent decades, invasive fungal infections have been increasing significantly, contributing to high incidences and mortality in immunosuppressed patients. *Candida albicans* (*C. albicans*) is the most prevalent opportunistic fungal pathogen in humans that can cause severe and often fatal bloodstream infections. Current antifungal agents have several limitations, including that only a small number of classes of antifungals are available, certain of which have severe toxicity and high cost. Moreover, the emergence of drug resistance is a new limitation to successful patient outcomes. Therefore, the development of antifungals with novel targets is an essential strategy for the efficient management of *C. albicans* infections. It is widely recognized that ion homeostasis is crucial for all living cells. Many studies have identified that ion-signaling and transduction networks are central to fungal survival by regulating gene expression, morphological transition, host invasion, stress response, and drug resistance. Dysregulation of ion homeostasis rapidly mediates cell death, forming the mechanistic basis of a growing number of compounds that elicit antifungal activity. Most of the potent antifungals have been widely used in the clinic, and certain of them have low toxicity, meaning that they may be expected to be used as antifungal drugs in the future. Hence, we briefly summarize the homeostasis regulation of several important ions, potential antifungal targets based on these ion-signaling networks, and antifungal compounds based on the disruption of ion homeostasis. This summary will help in designing effective drugs and identifying new targets for combating fungal diseases.

## Introduction

Invasive fungal infections are on the rise around the world, in parallel with increasing populations of immunosuppressed individuals; overprescription of chemotherapeutics, antifungal agents, and steroids; and extensive use of catheters, as well as other medical implants (Tobudic et al., 2012). *C. albicans* is the most widespread opportunistic fungal pathogen in the human body, causing mucosal, and systemic infections. More importantly, the mortality rate of bloodstream infections caused by *Candida* species was reported to be as high as 40–60% among immunosuppressed and hospitalized patients (Tobudic et al., 2012; Sun et al., 2015). *C. albicans* is polymorphic and capable of undergoing reversible morphological transitions between yeast, pseudohyphal, and hyphal growth forms. The hyphal form plays key roles in the infection process, and is coupled with many virulence factors including adherence and secretion of hydrolases (Vila et al., 2017). Biofilm production is related to a high level of antifungal resistance and easily occurs in host tissues, prostheses, and indwelling medical devices (Silva et al., 2017). Fungal-selective targets are insufficient due to the fact that most eukaryotes share similar metabolic pathways and essential cellular machinery with humans (Zhang et al., 2012). At present, antifungal agents are limited to three major classes: the polyenes, which bind fungal cell membrane ergosterol leading to cell lysis; azoles that inhibit ergosterol biosynthesis; and echinocandins that inhibit fungal (1,3)-β-d-glucan cell wall biosynthesis. Although echinocandins have a good safety profile, several of the traditional antifungals, such as itraconazole, voriconazole, and amphotericin B, possess severe toxicity (Zavrel and White, 2015). As a consequence of the wide use of antifungal agents, drug resistance of *C. albicans* is increasing, which poses a serious threat to antifungal therapy. Therefore, exploring effective antifungal agents with novel drug targets is urgently needed to cope with the challenges that the antifungal area faces (Guo X. et al., 2008).

**Figure 1:**
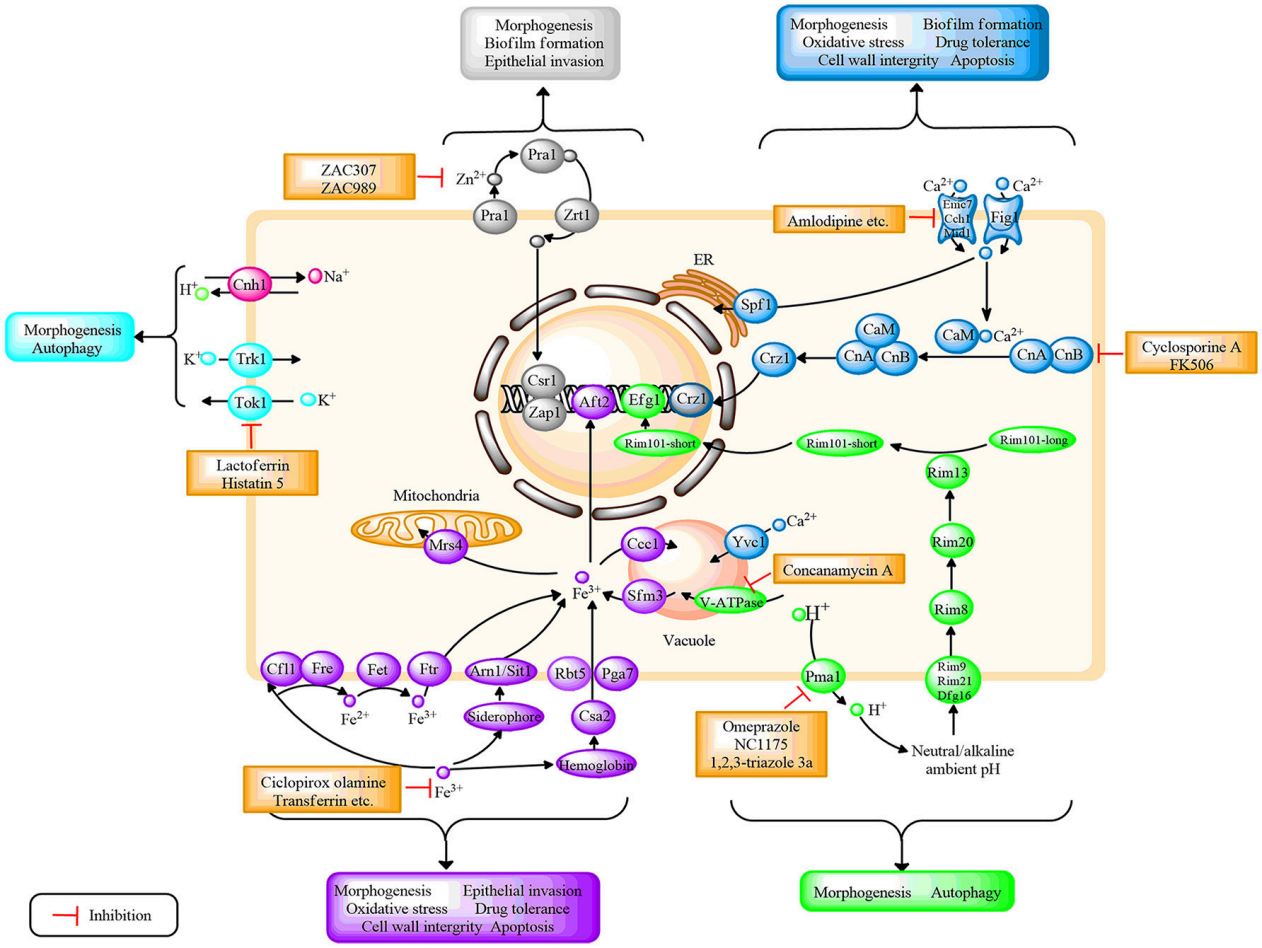
Schematic diagram depicting the regulation of different ion systems, their role in fungal growth, as well as the potential antifungal targets based on ion signaling pathways in *C. albicans*. Different ions correspond to different colors, H^+^(green); Ca^2+^(mazarine); Fe^3+^(purple); Zn^2+^(gray); K^+^(blue); and Na^+^ (pink). CaM, calmodulin; CnA, the a subunit of calcineurin; CnB, the b subunit of calcineurin. Further details are provided in the text.

**Figure 2:**
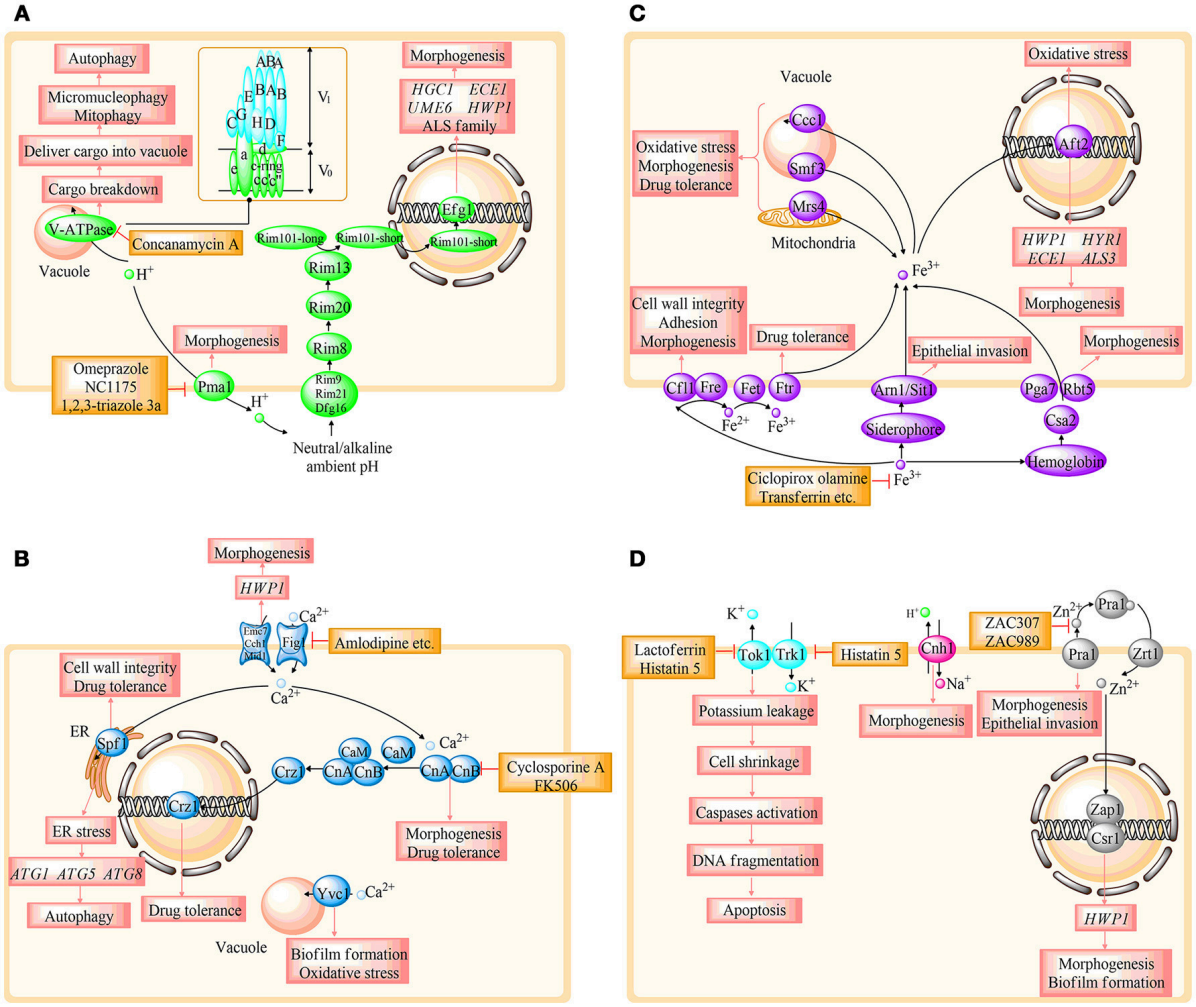
Overview of the relationship between the specific protein and fungal survival, tolerance, and virulence in different ion regulating systems. **(A–C)** represent H^+^, Ca^2+^, and Fe^3+^ homeostasis, respectively. **(D)** involves the homeostasis of Zn^2+^, K^+^, and Na^+^. *HGC1, ECE1, UME6, HWP1, HYR1*, and *HYR1*, hypha-specific genes; ALS, agglutinin-like sequence; CaM, calmodulin; CnA, the a subunit of calcineurin; CnB, the b subunit of calcineurin; *ATG*, autophagy-related genes. The details are shown in the text.

Ions play a vital role in various living organisms. In *C. albicans*, ions could participate in membrane potential maintenance, cell volume regulation, cofactors formation of multiple enzymes, proliferation, and apoptosis (Yun and Lee, 2017). Additionally, ionic signal transduction network is a central cellular pathway that has received extensive attention in recent years. Moreover, studies identified that ion homeostasis was closely connected with oxidative stress response, morphogenesis, antifungal drug resistance, cell wall integrity, and invasive growth in *C. albicans*, which reveal the potential for defining novel drug targets (Yu et al., 2014c; Loboda and Rowinska-Zyrek, 2017; Yun and Lee, 2017). Several studies have illustrated that dysregulation of ion homeostasis could rapidly mediate fungal growth and virulence (Davis, 2009; Lew, 2011). Therefore, strategies that target or manipulate ion homeostasis regulation may pave the way for novel antifungal agents. In the following, we primarily review the genes, proteins, and enzymes involved in the regulation of ion homeostasis, including hydrogen (H^+^), calcium (Ca^2+^), iron (Fe^3+^), zinc (Zn^2+^), potassium (K^+^), and sodium (Na^+^) in *C. albicans*. In addition, the potential antifungal targets based on ion homeostasis are summarized. In this review, we provide recent advances in this field, and the summary is presented in the form of figures and tables. Figure 1 illustrates the ion homeostasis regulatory pathways and potential antifungal targets on the basis of ion signal networks. Figure 2 highlights the link between specific ion homeostasis and fungal survival. Table 1 illuminates the compounds that exhibit antifungal activity by disrupting ion homeostasis.

**Table 1:** Antifungal activity of compounds against *Candida albicans* by ion disruption.

**Compounds**	***Candida albicans***	**Test parameter**			**MIC**	**Antifungal mechanisms or targets**	**References**
		**Medium**	**Temperature (°C)**	**Time (h)**			
**H**^+^ **homeostasis**							
1,2,3-triazole 3a	FLC^R^ *C. albicans* (15.9)	–	37	24	MIC = 25 μg/mL	Pma1 inhibitor	Irfan et al., 2017
NC1175	*C. albicans* (ATCC90028)	RPMI 1640	35	48	MIC_80_ = 1.66 μg/mL	Interact with Pma1	Manavathu et al., 1999
Concanamycin A	*C. albicans* DAY185	YPD	–^a^	–	MIC = 1.7 μg/mL	Inhibit V-ATPase-c-ring	Okoli et al., 2009
**Ca**^2+^ **homeostasis**							
EDTA	*C. albicans* (*n* = 3)	SDA	37	24	MIC = 0.625 mg/mL	Calcium chelators	Ates et al., 2005
EGTA	*C. albicans* (*n* = 3)	SDA	37	24	MIC = 20 mg/mL	Calcium chelators	Ates et al., 2005
Verapamil	*C. albicans* (*n* = 4)	RPMI 1640	37	24	–	Calcium channel blockers	Yu et al., 2014a
Beauvericin	*C. albicans* (SC5314)	RPMI 1640	–	–	MIC_90_ = 8 μg/mL	Elevate intracellular calcium	Tong et al., 2016
Silibinin	*C. albicans* (ATCC90028)	YPD	28	48	MIC = 40 μM	Disrupt calcium homeostasis in cytoplasm and mitochondria	Yun and Lee, 2016
**Fe**^3+^ **homeostasis**							
Ciclopirox Olamine	*C. albicans* (SC5314)	RPMI 1640	37	–	MIC_80_ = 2.0 μg/mL	Iron chelators	Niewerth et al., 2003
Geraniol	*C. albicans* (*n* = 8)	YPD	30	48	MIC_80_ = 225–250 μg/mL	Disrupt iron homeostasis	Singh et al., 2016
EMAC	FLC^R^ *C. albicans* (25)	RPMI 1640	35	72	MIC = 0.19–3.12 μg/mL	Iron chelators	Meleddu et al., 2016
Transferrin	*C. albicans* (SC5314)	RPMI 1640	35	20	MIC = 60 μg/mL	Sequester iron and disrupt membrane potential	Lin et al., 2014
**Zn**^2+^ **homeostasis**							
ZAC307	*C. albicans* (SC5314)	YPD	30	24	MIC_50_ = 0.6 μg/mL	Zinc chelators	O'Hanlon Cohrt et al., 2018
ZAC989	*C. albicans* (SC5314)	YPD	30	24	MIC_50_ = 0.4 μg/mL	Zinc chelators	O'Hanlon Cohrt et al., 2018
**K**^+^ **homeostasis**							
Histatin 5	*C. albicans*(*n* = 4)	YPD	30	24	MIC = 4–16 μg/mL	Interact with Trk1 and Tok1	Baev et al., 2003; Han et al., 2016
Cassia spectabilis	*C. albicans*	RPMI 1640	37	24	MIC = 6.25 mg/mL	Potassium leakage	Torey et al., 2016
Chlorogenic acid	*C. albicans* (ATCC 90028)	YPD	28	15	MIC = 320 μg/mL	Potassium leakage	Yun and Lee, 2017
Lactoferrin	*C. albicans* (ATCC 10231)	SDB	30	20	–	Potassium leakage through Tok1	Andrés et al., 2008

*MIC, minimum inhibitory concentration; YPD, yeast–polypeptone–glucose broth; SDA, Sabouraud dextrose agar; SDB, Sabouraud dextrose broth; FLC^R^, fluconazole-resistant; ^a^Indicates not mentioned in the reference*.

## H^+^ homeostasis and potential antifungal targets

### H^+^ homeostasis regulation

The pH of a solution refers to the negative logarithm of the H^+^ concentration.(Orij et al., 2011) It is widely recognized that the tight control of pH is a necessary requirement for the viability of all eukaryotic cells. Different organelles should maintain at distinct pH for multiple cellular events involving protein conformation, pH gradient establishment, and the regulation of enzymes activity (Kane, 2016). Fungi have evolved a number of strategies to accommodate its need of maintaining stable pH in response to rapid metabolism and a dramatically changing extracellular environment (Kane, 2016).

The pH homeostasis is primarily controlled by three types of components including pumps, exchangers, and buffers in all viable cells (Casey et al., 2010). As shown in Figure 2A, Pma1 (plasma membrane H^+^-ATPase) and V-ATPase are key regulators of intracellular pH that utilize the energy from ATP hydrolysis to transport proton out of the cytoplasm (Orij et al., 2011). Pma1 is thought to be the dominant proton pump of cytosolic pH, which belongs to the P-type H^+^-ATPase (Serrano et al., 1986). The proton pump is a 100-kD electrogenic, polytopic integral membrane protein with the N- and C- terminal transmembrane domains in the cytoplasm (Kane, 2016; Manzoor, 2016). Pma1 plays a crucial role in numerous ways such as creating the transmembrane electrochemical proton gradients that are necessary for nutrients and ions uptake and regulating cell growth and dimorphism in *C. albicans* (Khan et al., 2012; Kane, 2016). V-ATPase is another master regulator of pH that is highly conserved among all eukaryotic cells, and it is able to acidify intracellular organelles involving vacuole, endosomes, and Golgi apparatus (Kane, 2016). The substructure of V-ATPase is also illuminated in Figure 2A. This multisubunit enzyme is consisted of 14 subunits and divided into two domains. V_1_ complex, the peripheral membrane subunits responsible for hydrolyzing ATP, is making up of eight subunits from V_1_A to V_1_H (Olsen, 2014). V_0_ domain is the integral membrane proteins acting as a proton transporter, which contains six subunits known as a, c, c′, c”, d, e (Raines et al., 2013; Olsen, 2014; Kane, 2016). V_0_a is the only subunit composed by two homologs, with Vph1 located on the vacuole, whereas Stv1 is cycling between Golgi and endosomes (Finnigan et al., 2012). H^+^ gradients established by V-ATPase are essential for a diverse range of cellular functions such as in vacuole acidification, degradation of enzyme function, sequestration of toxic metal ions, receptor–ligand binding, and cargo sorting during endocytic and secretory pathways (Poltermann, 2005; Zhang and Rao, 2012; Olsen, 2014). Due to the prime regulation of Pma1 and V-ATPase cooperatively, intracellular pH could maintain the dynamic homeostasis, which ensures the normal development of cells.

### Potential antifungal targets related to H^+^ homeostasis

In *C. albicans*, optimum pH has been implicated in various virulence pathways involving a dimorphic switch and proteinases and lipases secretion (Patenaude et al., 2013; Raines et al., 2013). As the primary regulator of cytosolic pH, Pma1 could affect the dimorphic transition, which is essential for the host invasion and tissue damage (Kaur and Mishra, 1991; Seto-Young et al., 1997). Simminder Kaur et al. reported that *C. albicans* failed to form germ tubes and hyphae in the presence of Pma1 inhibitor orthovanadate (Kaur et al., 1988; Stewart et al., 1989). In addition, Pma1 plays a pivotal role in excreting protons to acidify the external environment, thereby, activating acid-activated proteases and lipases, which facilitates the penetration of *C. albicans* into host cells (Perlin et al., 1997). Patricia Soteropoulos et al. demonstrated that antagonists of plasma membrane H^+^-ATPase might exhibit broad-spectrum activity for the high similarity of sequence among these enzymes in diverse pathogenic fungi (Soteropoulos et al., 2000). Therefore, we can conclude that Pma1 could be developed as a surface-mediated and broad-spectrum antifungal target with new mechanisms for its unique structure and function (Monk et al., 2005; Manzoor, 2016).

V-ATPase is a critical pH regulator that participates in multiple virulence-related processes, including stress response, biofilm formation, morphology transition, host tissue invasion, and host immune response in *C. albicans* as shown in Figure 2A (Zhang and Rao, 2012; Patenaude et al., 2013; Raines et al., 2013). The pH gradient created by V-ATPase is required for the secretion of numerous virulence-related proteins, such as aspartyl proteases, lipases, adhesions, and invasins, in the secretory pathway, which assists *C. albicans* in the invasion and colonization to host cells (Zhang and Rao, 2012). In addition, V-ATPase is needed for the autophagic degradation processes that require an acid lumen environment to stimulate the enzymes responsible for various cargos degradation. Dalibor Mijaljica et al. speculate that functional V-ATPase is indispensable for the final step of autophagic processes between cargo breakdown and delivery into the vacuolar in micronucleophagy and mitophagy autophagy pathways (Yu et al., 2008; Kanki et al., 2009). In addition to the virulence-related roles of V-ATPase mentioned earlier, further studies have been conducted to reveal the specific function of V-ATPase subunits that associate with *C. albicans* virulence. V_0_a subunit is composed of Vph1 and Stv1; disruption of Vph1 could result in reduced activity of aspartyl protease and lipase, as well as filamentation defects (Raines et al., 2013; Rane et al., 2014). Moreover, Cassandra Patenaude et al. identified that Stv1 was associated with the secretion of adhesion-related proteins (Patenaude et al., 2013). V_1_B subunit is encoded by *VMA2*, and inhibition of the expression of *VMA2* leads to poor tolerance in oxidative response, temperature response, and stress response (Kane, 2006; Rane et al., 2014). *VMA3* encodes V_0_c subunit, and it is required for hyphae formation (Rane et al., 2013; Olsen, 2014). No orthologs of V_0_c' subunit exist in mammals; thus, this fungal-specific subunit possesses great potential to make it a desirable target for antifungal drug discovery (Raines et al., 2013; Rane et al., 2013). Moreover, *VMA5* is responsible for encoding the V_1_C subunit, which could not only affect hyphae formation and degradative enzymes secretion, but also be involved in the regulation of calcium concentration and maintenance of intracellular reactive oxygen species (ROS) (Zhang et al., 2017). Consequently, V-ATPase could serve as selective targets for antifungal drug development.

In addition to the intracellular pH, the ambient pH is another crucial factor that has a great impact on the morphological transition of *C. albicans* (Davis et al., 2000a). *C. albicans* is generally present in yeast form in acidic conditions, while in hyphae form under alkaline or neutral conditions, and this pH-response pathway is mediated by the transcription factor Rim101 (Davis, 2009; Cornet and Gaillardin, 2014). As depicted in Figure 2A, Dfg16, Rim9, and Rim21 are three receptor proteins located on the plasma membrane that act as environmental pH sensors. With the help of Rim8 and Rim20, the pH-changing signal could transmit to Rim13, which cleaves Rim101 precisely by identifying the enzymatic site on Rim101. Short-Rim101 is the active form that induces *EFG1* expression, regulating hyphae-specific genes such as *HGC1, ECE1, UME6, HWP1* as well as the agglutinin-like sequence (ALS) family, which is essential for adherence, biofilm formation, and host cell invasion (Fan et al., 2013; Cornet and Gaillardin, 2014). Dana Davis et al. confirmed that Rim101 pH response pathway could not only induce hyphae formation, but is also involved in virulence and pathogenicity *in vivo* (Davis et al., 2000a,b). Muriel Cornet et al. have demonstrated that all mutants in this pathway showed hypersensitivity to triazoles (fluconazole, voriconazole, and posaconazole) (Cornet and Gaillardin, 2014). Given that the Rim101 pH response pathway is essential for virulence, we can conclude that it is possible to exploit the Rim101 pH response pathway for new antifungal strategies.

### Antifungal agents based on H^+^ homeostasis

H^+^ homeostatic pathways are key regulators in *C. albicans* pathogenesis. Table 1 shows certain natural and synthetic compounds that exhibit an inhibitory effect against *C. albicans* by disrupting H^+^ homeostasis. Several studies demonstrated that the proton pump inhibitor, omeprazole, could inhibit *C. albicans* cell growth by restraining the activity of Pma1 (Monk et al., 1995; Perlin et al., 1997; Seto-Young et al., 1997). Activated lansoprazole is a novel benzimidazole proton pump inhibitor, which assists in the inhibition of hyphal growth (Monk et al., 1995; Perlin et al., 1997; Biswas et al., 2001). Furthermore, NC1175 and 1,2,3-triazole 3a are synthetic compounds that exhibit inhibitory activity against *C. albicans* by restraining the acidification of external medium with an minimum inhibitory concentration (MIC) of 25 μg/mL (Manavathu et al., 1999; Irfan et al., 2017). Additionally, concanamycin A, the most commonly used V-ATPase inhibitor, displays antifungal properties especially against *C. albicans*, and the MIC of concanamycin A is 25 μg/mL (Okoli et al., 2009). The accumulating knowledge may provide a novel avenue for antifungal drug discovery.

## Ca^2+^ homeostasis and potential antifungal targets

### Ca^2+^ homeostasis regulation

Calcium is one of the essential divalent ions, which is necessary for the growth of various eukaryotes (Cyert and Philpott, 2013). Furthermore, Ca^2+^ is a ubiquitous messenger that participates in the translation between various developmental signals and specific cellular responses. To serve its signaling function, a low cytosolic Ca^2+^ concentration must be maintained between 50 and 200 nM, which is achieved by the cooperation of various Ca^2+^ exchangers, pumps, and channels under specific conditions. Disruption of calcium homeostasis will lead to the growth defect and even cell death of all organisms (Liu et al., 2015).

Calcium cell survival (CCS) pathway (as described in Figure 2B) is the major calcium-signaling pathway that mediates cell survival under various environmental stimulates. The CCS pathway consists of four functional proteins, including the plasma membrane Ca^2+^ channel-containing Cch1 and Mid1, calmodulin (CaM), and calcineurin (CN). In the CCS pathway, environmental Ca^2+^ is first transferred into the cytoplasm through the calcium influx channel, the increased Ca^2+^ concentration results in the activation of CaM followed by the activation of calcium/CaM -dependent phosphatase CN. The transcription factor Crz1 is dephosphorylated by CN, leading to the translocation of Crz1 from the cytoplasm to the nucleus, which activates the expression of the various calcium-related genes (Thewes, 2014). Yeasts possess both high- and low-affinity systems for Ca^2+^ influx through the plasma membrane. The high-affinity calcium influx system (HACS) consists of three proteins containing Mid1, Cch1, and Ecm7. While the low-affinity calcium influx system (LACS) is the second and less well-defined Ca^2+^ influx system, displaying a 16-fold lower Ca^2+^ affinity than HACS, and FIG.1 is the core component of LACS (Harren and Tudzynski, 2013). In yeast, CaM is a ubiquitous and necessary protein that is required for the activation of CN. The CN is a highly conserved protein phosphatase, which consists of two subunits involving the catalytic subunit (encoded by *CNA1, CNA2*) and the regulatory subunit (encoded *CNB1*) (Cyert and Philpott, 2013).

In addition to the CCS pathway, there are certain components that are related to the Ca^2+^ release from cellular calcium pools. The vacuole is the primary Ca^2+^ storage organelle, which contains nearly 90% of the total intracellular Ca^2+^, and the effect of sequestrating intracellular Ca^2+^ mainly exerts through the H^+^/ Ca^2+^ exchanger Vcx1, as well as the P-type Ca^2+^-ATPase Pmc1. Vcx1 plays the dominant role in short-term Ca^2+^ dynamics,which utilizes the H^+^ gradient produced by V-ATPase to transport Ca^2+^ into the vacuole coupled with the H^+^ efflux into the cytoplasm. Furthermore, Yvc1 is a member of the transient receptor potential (TRP) channels with the primary function of mediating the release of Ca^2+^ stored in the vacuole (Cyert and Philpott, 2013; Yu et al., 2014c,d). Moreover, the endoplasmic reticulum (ER) is the major organelle responsible for protein folding and secretion; the Ca^2+^ homeostasis of the ER is primarily governed by the P-type ATPase Spf1, whose homolog is ScCod1/ScSpf1 in *Saccharomyces cerevisiae* (Yu et al., 2013b, 2015). In conclusion, diverse elements maintain the Ca^2+^ homeostasis cooperatively, aiming to regulate the growth and metabolism of cells.

### Potential targets related to ca^2+^ homeostasis

Calcium, as a critical second messenger in eukaryotic cells, plays a direct role in the signal transduction and cellular responses. Moreover, calcium is closely related to the regulation of stress response, morphogenesis, drug tolerance, and cell wall integrity in *C. albicans*. Thus, calcium is of key importance to maintain the optimal level of intracellular Ca^2+^ (Yu et al., 2012a; Li and Sun, 2016). The CCS pathway is the central calcium transduction pathway, which is tightly connected with the virulence of *C. albicans*. Qilin Yu et al. confirmed that Cch1 and Mid1 were involved in the formation and maintenance of hyphae, oxidative stress response, and invasive growth in *Candida albicans*. Mutants deleted of *CCH1* and *MID1* showed reduced virulence in a murine model of infection. Moreover, mutants defected of Cch1 or Mid1 function were highly hypersensitive to azoles, indicating that CCS pathway is crucial for cell survival in azole therapies (Yu et al., 2012a). Xiaohui Ding et al. proved that Ecm7, a regulator of HACS, functions in the maintenance of intracellular redox homeostasis and hyphal formation in *C. albicans*. The mutants lacking *ECM7* resulted in ROS accumulation in the presence of H_2_O_2_ and decreased expression of the hyphae-specific gene *HWP1* (Ding et al., 2013). Calcineurin is highly associated with virulence and drug resistance in a diverse group of pathogenic fungi, such as *Cryptococcus neoformans, C. albicans*, and a number of plant fungal pathogens (Juvvadi et al., 2017). Many studies have shown that CN and its downstream target Crz1 are responsible for the transition between morphological states, stress responses, cell wall integrity, and tolerance to antifungal agents, such as azoles, terbinafine, and echinocandins (Sanglard et al., 2003). The CN plays a critical role in cell wall integrity pathways, which contributes to cell survival in the host environment (Sanglard et al., 2003; Juvvadi et al., 2017). Moreover, Thewes et al. confirmed that CN is essential in a murine model of infection, whereas Crz1 has a moderate effect on virulence (Karababa et al., 2006; Thewes, 2014). In conclusion, the CCS pathway provides attractive targets for the exploitation of potent antifungal agents.

In addition to the CCS pathway, regulation of calcium concentrations by intracellular organelles also has a profound effect on *C. albicans* survival. Spf1, the P-type ATPase located on the ER, is associated with autophagy, cell wall integrity, drug resistance, and biofilm formation in *C. albicans* (Yu et al., 2012b, 2013b, 2015). Qilin Yu et al. speculate that there is a possible correlation between autophagy and ER stress, and the deficiency of Spf1 function leads to the disorder of calcium homeostasis and ER stress, which leads to the increased autophagic activity and upregulation of autophagy-related (*ATG*) genes including *ATG1, ATG5*, and *ATG8* (Yu et al., 2015). In addition, Qilin Yu et al. demonstrated that *spf1*Δ/Δ mutant showed hypersensitivity to cell wall stress and abnormal cell wall organization, suggesting that the mutant is deficient in cell wall integrity, which is essential for the virulence of *C. albicans*. Moreover, the *spf1*Δ/Δ mutant showed significant hyphae and biofilm formation defects in hyphae-inducing media (Yu et al., 2012b). Yvc1 is a member of vacuolar locating TRP channel family, which plays a significant role in fighting against oxidative stress (Yu et al., 2014d). Additionally, Qilin Yu et al. suggested that disruption of Yvc1 function resulted in attenuated virulence of mouse models and human epithelial cells. Yvc1 is necessary for hypha-associated gene expression during hyphal elongation and maintenance, while it is dispensable for hyphal formation (Yu et al., 2014c). In summary, disruption of Ca^2+^ homeostasis should improve the development of antifungal agents and provide alternatives for the treatment of *C. albicans* infection.

### Antifungal agents based on ca^2+^ homeostasis

Studies revealed that disruption of Ca^2+^ homeostasis by specific compounds not only regulates cell survival, but also influences critical functions for the pathogenesis of *C. albicans*. Several reports have shown that certain agents could inhibit the growth of *C. albicans* by disrupting Ca^2+^ homeostasis, as shown in Table 1. Ethylenediaminetetraacetic acid (EDTA) and ethylene-*bis*(oxyethylenenitrilo)tetraacetic acid (EGTA) are two calcium chelators that possess antifungal activity contributing to their property of calcium chelating (Ates et al., 2005). Moreover, Yaojun Tong et al. identified that beauvericin had fungicidal activity *in vitro* by elevating intracellular calcium and ROS, and the MIC_90_ of beauvericin was 8 μg/mL (Tong et al., 2016). Particularly, mitochondrial Ca^2+^ overload plays a crucial role in the process of apoptosis. Silibinin could induce the activation of pro-apoptotic factors of *C. albicans* (Yun and Lee, 2016). Traditional antifungal agents could affect cell survival directly by interfering with ergosterol or the cell wall. Recent studies have shown that some drugs can produce antifungal activity by inhibiting virulence-related factors, such as adhesion, biofilm formation, and so on. It is worth noting that certain non-antifungal agents have significant synergetic effects when combined with traditional antifungal agents. Priya Uppuluri et al. reported that *C. albicans* biofilms are hypersensitive to CN inhibitor–fluconazole combinations. Cyclosporine A (CsA) and tacrolimus (FK506) are immunosuppressive drugs, which inhibit the activity of CN and possess dramatic synergistic effect against resistant *C. albicans* along with fluconazole treatment. The mechanisms of the synergistic effects are related to the inhibition of several virulence factors including cell adhesion, hyphal formation, and the downregulation of *ALS3, HWP1, CDR1, MDR, ERG11* gene expressions (Juvvadi et al., 2017). Moreover, several studies have demonstrated that verapamil showed an inhibitory effect against *C. albicans* when used alone or in combination with fluconazole, and the synergistic effect was closely associated with biofilm development and adherence (Yu et al., 2013a, 2014a). Additionally, Shuyuan Liu et al. identified that several calcium channel blockers (amlodipine, nifedipine, benidipine, and flunarizine) showed a significant synergetic effect on resistant *C. albicans* when combined with fluconazole. The expressions of *CNA1, CNB1*, and *YVC1* were downregulated when fluconazole was combined with amlodipine. Although amlodipine has certain side effects, it is currently used as an antihypertensive drug in clinical practice. Despite its limitation, the result of these data will provide new insights for the development of novel antifungal drugs with novel mechanisms (Liu et al., 2016). Studies have demonstrated that the antiarrhythmic drug amiodarone showed a synergistic effect against *C. albicans*, and the antifungal activity of amiodarone is mediated by the disruption of calcium homeostasis. Zhang et al. evaluated the clinical potential of combining fluconazole and amiodarone in treating fungal infections in a murine Candidiasis model. The microbial burden of *C. albicans* in kidneys was significantly reduced in the combination group (Gupta et al., 2003; Guo Q. et al., 2008; Zhang et al., 2010). In conclusion, blocking the calcium signaling pathway by specific inhibitors is tightly associated with cell survival, and further understanding of the calcium-related mechanisms of virulence and pathogenicity should provide novel ideas to the development of antifungal agents.

## Fe^3+^ homeostasis and potential antifungal targets

### Fe^3+^ homeostasis regulation

Iron is an essential nutrient as well as the most abundant trace metal in all eukaryotic cells. Iron is capable of converting between ferrous and ferric forms (Bairwa et al., 2017). The redox characteristic of iron plays a crucial role in carrying out vital cellular functions in many central metabolic pathways such as DNA synthesis, respiration, electron transport chains, oxygen transport/storage, and tricarboxylic acid cycle (Bairwa et al., 2017). However, iron can be toxic as it catalyzes the production of ROS that can severely damage biological molecules including nucleic acids, proteins, and lipids (Balhara et al., 2016; Malavia et al., 2017; Mamouei et al., 2017; Gerwien et al., 2018). Hence, all eukaryotes have developed exquisite iron-acquisition strategies to maintain the dynamic balance between cell growth and cytotoxicity.

The majority of iron in host cells is bound to iron-binding proteins such as hemoglobin, ferritin, and transferrin, with virtually no free iron in circulation. In the host environment, fungi such as *C. albicans* have evolved a sophisticated iron uptake regulatory network as described in Figure 2C (Malavia et al., 2017; Gerwien et al., 2018). There are three significant iron acquisition systems, including reductive iron uptake system, siderophore uptake system, and hemoglobin uptake system in *C. albicans*. Reductive iron uptake system is especially crucial in non-siderophore-producing *C. albicans*, which contributes to its growth and virulence (Gerwien et al., 2016). The bound Fe^3+^ that is extracted from ferric salts and ferric chelates is first reduced to Fe^2+^ via ferric reductases Cfl1 and Fre. Fe^2+^ is then oxidized by permease-coupled multicopper ferroxidases Fet, followed by Fe^3+^ transport into the intracellular space via the high-affinity permeases Ftr (Bairwa et al., 2017). Cfl1 was the first identified ferric reductase in *C. albicans*, which could rescue the growth defect in *S. cerevisiae fre1* mutant (Xu et al., 2014b; Yu et al., 2014b). Moreover, the ferroxidase is encoded by five genes(*FET3, FET31, FET33, FET34, FET99*), and four permease genes are annotated as *FTR1, FTR2, FTH1*, and *FTH2*. Researchers have shown that *C. albicans* possesses multiple high-affinity iron transporters formed by a permease associated with a ferroxidase specifically (Muzzey et al., 2013; Mamouei et al., 2017). The second iron uptake system is the siderophore uptake system. Siderophore, as the central part of the siderophore uptake system, is a class of small molecules secreted by bacteria and fungi, which is capable of binding with extracellular Fe^3+^ with extremely high affinity (Gerwien et al., 2018). The iron transport process is followed by the combination of the Arn1/Sit1 siderophore transporter. *C. albicans* could utilize the siderophores secreted by other species although they are not siderophore producers (Bairwa et al., 2017; Gerwien et al., 2018). Hemoglobin uptake system is another critical iron uptake system, which is an essential iron source for *C. albicans*. Approximately 80% of iron inside the mammalian host is sequestered within host iron-binding proteins, and this is the reason why hemoglobin uptake system is necessary for iron acquisition in *C. albicans*. It has been speculated that the members of the *C. albicans* heme-receptor protein family possess the common in several fungal extracellular membranes (CFEM) domain involving Csa2, Pga7, and Rbt5, as illustrated in Figure 2C. The Csa2 functions as a secreted hemophore to deliver the hemoglobin to other CFEM proteins, such as Rbt5 and Pga7 (Nasser et al., 2016). Both Rbt5 and Pga7 are able to efficiently extract heme from hemoglobin, while Rbt5 shows a higher affinity and a higher amount (Malavia et al., 2017). Iron acquisition from the host environment is precisely regulated by the iron uptake systems, which ensures *C. albicans* cell growth.

In addition to the iron uptake systems, intracellular iron homeostasis also requires the regulation of several elements inside the cell. As shown in Figure 2C, the Mrs4–Ccc1–Smf3 pathway is composed of three iron transporters located on the intracellular organelles and plays a crucial role in the regulation of intracellular iron homeostasis (Xu et al., 2014a). Mrs4, the mitochondrial carrier proteins, facilitates the transport of iron across the mitochondrial inner membrane, as well as maintaining the mitochondrial morphology (Froschauer et al., 2009). Ccc1 is an iron transporter located at the vacuolar, and is involved in the sequestration of iron from the cytosol to the vacuole at the time when the media contains adequate amounts of iron (Li et al., 2001). Smf3 is the homolog of the Nramp family that helps to mobilize vacuolar stores of iron (Portnoy et al., 2000). Additionally, fungi have developed various iron-regulated systems to adjust cell requirements under diverse host environment. In *S. cerevisiae*, Aft1 and Aft2 are two iron-regulatory activators whose functions are involved in the transcription of iron uptake and metabolism-related genes (Goncalves et al., 2014). Ning Xu et al. have identified that *C. albicans* Aft2 is the ortholog of *S. cerevisiae* Aft1/Aft2 regulators, which is tightly associated with the expression of iron-related genes (Liang et al., 2010). We could summarize that the regulation of iron homeostasis is particularly sophisticated and plays a crucial role in *C. albicans* cell survival.

### Potential targets related to Fe^3+^ homeostasis

Several studies have implied that iron availability may play a significant role in signaling or facilitating the transition from commensal to pathogenic in *C. albicans*. The efficient iron acquisition composes necessary virulence factors and is involved in the invasion of the host epithelium and morphological transition (Spacek et al., 2005). The reductive iron uptake system in *C. albicans* is coordinated with adhesion, filamentation, antifungal drug resistance, cell wall integrity, and mitochondrial function (Jeeves et al., 2011; Yu et al., 2014b; Mamouei et al., 2017). The ferroxidases must cooperate with each other to support the full virulence. Fet shows an effect on cell growth, hyphal development, and virulence. Disruption of *FET99* results in a clear growth deficiency. Moreover, loss of *FET34* impedes the virulence of *C. albicans* in a mouse model of system infection, and the mutant exhibits a filamentous growth defect (Cheng et al., 2013; Mamouei et al., 2017). Cfl1 plays a significant role in the maintenance of the cell wall architecture, mitochondrial function, filamentous growth, and oxidative response in *C. albicans*. Disruption of *CFL1* results in ROS accumulation. Furthermore, Cfl1 dysfunction leads to the defect in cell wall integrity (Xu et al., 2014b; Yu et al., 2014b). Siderophore uptake system is mediated by Sit1/Arn1 and is required for the epithelial invasion and penetration caused by *C. albicans* infection (Heymann et al., 2002). The hemoglobin uptake system is the third host-iron assimilation pathway, which is expressed not only during mouse model infections, but also in candidemia patients. Kazuko Okamoto-Shibayama et al. suggested that hemoglobin is a source of iron, which has an impact on the hyphal morphogenesis in *C. albicans* (Okamoto-Shibayama et al., 2014).

The Mrs4–Ccc1–Smf3 (MCS) pathway plays a vital role in maintaining intracellular iron homeostasis and is closely related to cell survival. Ning Xu et al. have demonstrated that the mitochondrial dysfunction caused by the MCS pathway dysregulation had a tremendous effect on various physiological processes, such as oxidative stress response, cell-wall stability, colony morphology, and filamentous growth (Xu et al., 2012, 2014a). Aft2 is the most important regulator of iron metabolism-related genes, and it also shows an impact on virulence, invasive growth, oxidative response, and morphology transition. Deletion of *AFT2* leads to the accumulation of ROS and dramatically elevated superoxide dismutase activity under H_2_O_2_ treatment. Moreover, *aft2*Δ/Δ mutant also downregulates the transcript levels of hypha-specific genes involving *HWP1, HYR1, ECE1*, and *ALS3*, which have an effect on invasive growth in *C. albicans*. The information on iron homeostasis regulation sets the stage for a consideration of approaches to exploit novel antifungal targets.

### Antifungal agents based on Fe^3+^ homeostasis

The accumulating information provides opportunities to exploit iron acquisition for antifungal therapy, and new work highlights the development of iron chelators for therapeutic use alone or in conjunction with existing antifungal drugs. Table 1 lists the compounds that possess antifungal activity by disrupting iron homeostasis. Transferrin could restrict *C. albicans* growth *in vitro* due to its capacity of iron sequestration and membrane potential disruption, with an MIC_80_ of 2.0 μg/mL (Lin et al., 2014). Moreover, Markus Niewerth et al. identified that ciclopirox olamine could act as an iron chelator which in turn, influences iron metabolism and therefore reduces cell growth (Niewerth et al., 2003). Certain plant compounds and their derivatives also exhibit the properties of inhibiting the growth of *C. albicans* by disturbing iron homeostasis; these plant-derived compounds include eupolauridine and geraniol. Moreover, geraniol inhibits virulence factors that attribute to hyphal morphogenesis, biofilm formation, and cell adhesion (Meleddu et al., 2016; Singh et al., 2016; Tripathi et al., 2017). In addition, iron depletion in *C. albicans* with bathophenanthroline, disulfonic acid, and ferrozine as chelators enhanced its sensitivity to traditional antifungal agents (Prasad et al., 2006). In conclusion, compounds that block iron availability can effectively fight against *C. albicans*; this strategy represents a novel insight in the development of antifungal agents.

## Zn^2+^ homeostasis and potential antifungal targets

### Zn^2+^ homeostasis regulation

Zinc is the second-most abundant trace metal and is shown to be a second messenger that participates in various intracellular transduction signaling pathways. Zinc is indispensable in enzymes such as superoxide dismutases and metalloproteases that are crucial for the virulence and survival of fungal pathogens in host cells (Yike, 2011). Moreover, zinc-binding proteins are involved in transcription regulation via different zinc finger transcription factors in *C. albicans* (Loboda and Rowinska-Zyrek, 2017). However, because the concentration of free Zn^2+^ is as low as sub-nanomolar, excess zinc shows toxicity by binding to proteins inappropriately. Therefore, fungi have evolved sophisticated mechanisms to control intracellular zinc homeostasis tightly.

Zinc homeostasis regulation is shown in Figure 2D. The uptake of zinc from the host environment takes place mainly via two ZIP transporters in *C. albicans*. Zrt1 provides the high-affinity zinc uptake activity, while Zrt2 shows lower zinc affinity than Zrt1 (Kim et al., 2008). In addition to the Zrt membrane transporter, *C. albicans* is able to secrete an extracellular zinc-binding protein Pra1. This process is followed by the delivery to the Ztr1 transporter. The Pra1–Zrt1 zincophore transport system is more effective under zinc depletion and an alkaline environment (Gerwien et al., 2018). Moreover, zinc homeostasis is largely achieved by the expression of a zinc-related transcriptional factor. In yeast, Zap1 is the major zinc-sensing transcriptional factor that activates more than 80 genes' transcription including *ZRT1, ZRT2* in response to zinc deficiency (Wu et al., 2008; Gerwien et al., 2018). Kim et al. have demonstrated that Csr1 is another functional zinc-responsive transcription factor, which shows a high similarity to Zap1 according to the amino acid sequence analysis (Kim et al., 2008).

### Potential targets related to Zn^2+^ homeostasis

It is well-known that zinc takes part in various intracellular physiological and metabolic processes. In contrast, far less is known concerning how zinc homeostasis influences eukaryotic pathogens virulence. Several studies have suggested that zinc homeostasis plays diverse roles in the morphological transition, biofilm formation, and virulence of *C. albicans* (Figure 2D) (Lehtovirta-Morley et al., 2017). Zrt1 is associated with cell growth and *ZRT1* disruption results in growth defects in the neutral alkaline zinc-limited medium (Citiulo et al., 2012). Moreover, both Pra1 and Zrt1 were found to be upregulated in order to uptake adequate zinc in epithelial cells and liver infection models (Xu et al., 2015). Francesco Citiulo et al. suggested that the zincophore Pra1 mediates endothelial damage by scavenging host zinc because deletion of *PRA1* showed significantly shorter hyphae than the wide type (Citiulo et al., 2012). Previous studies have shown that Zap1 could govern proliferation in a pathway that operates in the invasive infection environment. In addition, Zap1 has also been identified as a hypha-formation regulator that governs the balance of yeast and hyphal cells in biofilms. Csr1 is the ortholog of Zap1 in *C. albicans*, which not only controls zinc homeostasis, but also contributes to the filamentation and biofilm elaboration. Kim et al. have demonstrated that the transcriptional level of the hypha-related gene *HWP1* was significantly inhibited in the *CSR1* deletion mutant (Kim et al., 2008). Taken together, zinc homeostasis provides a promising step in the advancement of antifungal drug development.

### Antifungal agents based on Zn^2+^ homeostasis

In recent years, the importance of zinc homeostasis has been highlighted due to its vital contribution to fungal pathogenesis and virulence. Researchers identified two novel zinc-attenuating compounds (ZACs) ZAC307 and ZAC989, which showed antifungal activity against *C. albicans* by chelating zinc *in vitro* (O'Hanlon Cohrt et al., 2018). ZAC307 and ZAC989 have a high-binding affinity for zinc. As shown in Table 1, the MIC_50_ of ZAC307 and ZAC989 are 0.6 and 0.4 μg/mL, respectively. Moreover, Karen et al. reported that ZAC307 and ZAC989 exhibited *in vivo* efficacy in a murine fungal kidney burden candidiasis model. In summary, this series of ZACs represents a potentially new class of antifungal agents, and interfering with fungal zinc-dependent processes represents a promising new approach to antifungal therapy.

## Others

### K^+^ homeostasis and cell survival

Potassium exists at higher concentrations (200–300 mmol/L) in *Candida* species, and the sufficient amount of potassium is essential for various cell functions, such as regulating cell volume and pH, compensating negative charge, and maintaining a membrane potential, and activation of key metabolic processes involving protein synthesis and stabilization (Ariño et al., 2010).

As shown in Figure 2D, Tok1 is the only certified K^+^-specific channel in Candida plasma membranes, which is activated by membrane depolarization and may be the principal voltage stabilization pathway in yeast cells. Although the sequence motifs of K^+^ channels are similar between fungi and mammals, Tok1 may also be regarded as attractive pharmacological targets because the fungal homologs of Tok1 possess the unique topological structure and gating mechanism (Loukin et al., 2002; Prole and Taylor, 2012).

Cell shrinkage is one of the most obvious features of apoptosis, and numerous studies have shown that the release of potassium is closely linked to the cell shrinkage for the reason that K^+^ is the major determinant of cell volume (Bortner and Cidlowski, 2007). Moreover, enhanced K^+^ efflux has been shown to mediate the downstream caspases activation and DNA fragmentation, which lead to apoptosis (Remillard and Yuan, 2004). As listed in Table 1, several studies have confirmed that some compounds can induce apoptosis of *C. albicans* by promoting the outflow of K^+^. Chlorogenic acid and *Cassia spectabilis* caused excessive potassium efflux, followed by apoptotic volume decrease, caspase activation, and DNA fragmentation (Torey et al., 2016; Yun and Lee, 2017). Andres et al. verified the role of potassium channels in the apoptosis of *C. albicans*. Cell death induced by human lactoferrin may be associated with a release of high concentrations of potassium through Tok1 channel (Andrés et al., 2008). Furthermore, studies have reported that Trk1 and Tok1 are critical transporters in the killing of *Candida* by the oral antimicrobial peptide, histatin 5 (Baev et al., 2003; Han et al., 2016).

### Na^+^ homeostasis and cell survival

K^+^ is essential in many important physiological roles as described earlier. In contrast, Na^+^, which shares similar atomic structures and properties with K^+^, shows toxicity at high concentration, and the toxicity is partly because of its substitute for K^+^ (Page and Di Cera, 2006). Therefore, the Na^+^ surplus must be effectively eliminated to maintain the minor concentration in the cytosol (Rodrígueznavarro, 2000). As described in Figure 2D, Cnh1 is the plasma membrane Na^+^/H^+^ antiporter, which utilizes the proton gradient to mediate the efflux of alkali metal cations and ensure the optimal intracellular level of them (Kinclova-Zimmermannova and Sychrova, 2007). Tuck-Wah Soong et al. verified that deletion of *CNH1* led to the retarded growth *in vitro* and a considerable delay in the infected mouse model, and the pathogenicity-related features of *CNH1* rendered the gene a potential target for developing anti-*Candida* agents (Soong et al., 2000).

## Conclusion

Infections caused by *C. albicans* have been increasing in recent years and can result in local or systemic infections with high morbidity and mortality. The increase of drug resistance within *C. albicans* makes it crucial to investigate novel antifungal targets further. It should be considered that ions are crucial in every cellular system, since they are involved in a wide variety of important metabolic processes and pathogenesis. Moreover, fungi have evolved an accurate regulatory uptake and detoxification system for essential ions such as hydrogen (H^+^), calcium (Ca^2+^), iron (Fe^3+^), zinc (Zn^2+^), potassium (K^+^), and sodium (Na^+^). Interfering with ion-dependent processes in fungi may be an effective approach to defeat these microorganisms. Recent studies have shown that some agents are able to inhibit *C. albicans* essential biological processes, such as growth and proliferation, as well as relevant pathological events, such as adhesion to host structures, evasion of host immune response, and the regulation of virulence factors. Moreover, further studies have shown that compounds disrupting ion homeostasis could exert antifungal effects or even reverse the *C. albicans* drug resistance. Although certain limitations still exist in the development of antifungal drugs based on ion homeostasis due to the highly conserved structure in humans, we still believe that it is of great value. On the one hand, a series of *in vivo* studies have shown that compounds based on ion homeostasis could increase the survival rate of murine or *Galleria mellonella* Candidiasis models when used alone or in combination with antifungal agents. On the other hand, the new mechanisms based on ion homeostasis may provide clues for the search of fungal-specific targets. There is no doubt that more studies are needed to explore how to target ion signaling pathways in fungi for antifungal therapies.

## Author contributions

YL wrote the review. SS, LS, CL, YG, and ML also contributed to the writing of this article. The authors are grateful for the suggestions of SS regarding the language and structure of the review.

### Conflict of interest statement

The authors declare that the research was conducted in the absence of any commercial or financial relationships that could be construed as a potential conflict of interest.
